# Performance Sex Differences in CrossFit^®^

**DOI:** 10.3390/sports10110165

**Published:** 2022-10-25

**Authors:** Petr Schlegel, Adam Křehký

**Affiliations:** Department of Physical Education and Sports, Faculty of Education, University of Hradec Kralove, Hradec Králové 500 03, Czech Republic

**Keywords:** gender, endurance, strength, competition

## Abstract

CrossFit^®^ has a unique standard for workout of the day for women and men. Scaling is used to set difficulty levels for women in CrossFit^®^ gyms and competitions. This type of scaling is applied for weightlifting (60–82% of men’s load); however, there are usually no differences in difficulty settings for gymnastics and monostructural metabolic conditioning. Performance analysis is essential for every sports discipline, and statistical data comparing men’s and women’s results from athletics, running, swimming, weightlifting, etc., are available. However, CrossFit^®^ lacks these statistics. The aim of our study was to analyze how the performances of men and women differed at the 2021 CrossFit Games^®^. Our sample comprised 40 female (age 27.8 ± 5.1) and 40 male participants (age 27.2 ± 3.7) competing in the Rx division. Data obtained from all events were analyzed using effect size and percentage. In 14 out of 15 events, men achieved better results than women. Even with the implementation of scaling, women’s results differed by 0.1–33.1% (effect size from small to large). Scaling for women is designed according to general strength and power differences; however, primarily because of anatomic and physiological differences, men attain better results. However, CrossFit Games^®^ events are always unique, and the events rarely repeat; therefore, our study does not provide firm conclusions. As our study is the first to compare CrossFit Games^®^ performance between the sexes, further research is needed.

## 1. Introduction

CrossFit^®^ is more than just an activity to keep you fit, it can be regarded as a sports discipline, community, or lifestyle. Since its establishment in 1990, CrossFit^®^ has been advocating for gender equality [1], and its approach questions traditional femininity and sex differences [2]. Its competitions also place importance on equal conditions, and the number of events for men and women competing is always equal.

CrossFit^®^ uses scaling to create optimal conditions for various age groups, adaptive athletes, or performance levels. Its WOD (workout of the day) was created with specific options for both men and women [3]. CrossFit^®^ comprises three individual and equally valued modalities: gymnastics (G), weightlifting (W), and monostructural metabolic conditioning (M). Women’s scaling applies mostly to W, which involves Olympic weightlifting, powerlifting, dumbbells, medicine balls, etc. The modalities of G (pull-up, handstand push-up, muscle-up, one-leg squat, etc.) and M (running, rowing, swimming, rope jumping, etc.) are almost always equal [4]. The same principle is used in competitions. However, exceptions are possible. In the last five years of the CrossFit Games^®^, exceptions were applied to a total of six events—four in M (number of calories) and two in G.

The CrossFit Games^®^ event has similar features to a championship. Over four days, athletes take part in multiple events that are similar to training for the general population of individuals who do CrossFit. However, the environments are different. Events take place “out of the gym”—in fields, stadiums, beaches, etc., and with less common equipment (paddleboard, mountain bike, “Pig”, etc.). The equipment and difficulty of movements in some events differ (e.g., handstand walking across parallel bars). Competition events are usually extremely variable in terms of their program duration and modalities. During the events, the G, W, and M modalities are either combined or only one is used [5]. The athletes have to show top-level performance in a broad spectrum of skills and abilities, such as maximum strength, strength endurance, and aerobic or anaerobic endurance. The aim is to find the “fittest on earth”. 

Researchers have conducted several studies concerning the important predictors for CrossFit^®^ performance. For instance, Dexheimer et al. [6] and Feito et al. [7] identified aerobic and anaerobic endurance as important parameters. However, their conclusions were based on a specific workout that did not meet the broad CrossFit^®^ requirements. Serafini et al. [5] analyzed benchmark performances from both sexes (the data were taken from the profiles of competitors) and compared deadlift, back squat, and snatch results. According to the level of the athletes, performance increases were comparable in both sexes; however, women reached approximately 65% of men’s load. Martínez-Gómez et al. [8] and Schlegel et al. [9] conducted research on the relationship between ranking in the CrossFit Open (first qualifying round for CrossFit Games^®^ and strength and endurance tests, and they determined back squat and Olympic weightlifting performance as key predictors for high ranking. However, CrossFit Games^®^ athletes have not been analyzed in terms of predictors and ranking. The relationship between exercise in the CrossFit Open and physiological fitness measures and self-reported fitness has been studied with a sample of amateur and (semi)professional CrossFit competitors [10]. Body-fat percentage and vastus lateralis cross-sectional area were key predictors. A medium to high positive correlation was found for VO_2peak_ in all workouts except weightlifting. 

Some researchers also included women in their study sample, and there were no differences in the analyses concerning men and women [6,7,10]. Tibana et al. [11] found comparable predictors (strength, specific muscle endurance test) for the CrossFit Open for both men (n = 11) and women (n = 6). For women, in contrast to men, VO_2max_ had a large positive correlation with event results. Significant sex differences emerged from correlation analyses between rankings and selected benchmark workouts [12]. Among men, no significant relationship was found in any workout; however, among women, variables such as the 400 m run and weights in the clean, jerk, and snatch events had a significant correlation to ranking. Despite this existing research, the information concluded in these studies is not sufficient to determine differences between men’s and women’s performances.

Physiological factors may be considerable when comparing performance results between men and women. Each sex has different anatomic and physiological predispositions for strength- and endurance-related performance. Men have more muscle mass; less fat mass; more type 2 muscle fibers; and higher levels of muscle glycogen storage, VO_2peak_, hemoglobin and red blood vessels, maximal anaerobic power, and testosterone and growth hormones [13,14]. Women have more type 1 muscle fibers, more effective beta-oxidation of fatty acids, less vascular occlusion during muscle work, lower central and local fatigability, higher muscle endurance, and higher levels of estrogen, progesterone, and luteinizing hormone [15,16,17]. However, both groups have shown comparable results in terms of movement economy [18]. 

Performance differences among men and women are apparent in all disciplines of CrossFit^®^. In Olympic weightlifting, women’s performance is around 64–80% of men’s; furthermore, in powerlifting, it is 61% [19,20]. In gymnastic exercises focused on the upper body, men performed 1.6–2.3 times more repetitions with the difference being 40–62% [21,22,23]. In short-distance (<20 min) endurance disciplines (running, rowing, swimming, mountain skiing, etc.), the difference is 6–11% [14,16,24]. 

Statistical data on performance differences between men and women are essential for every sports discipline—they provide considerable information about current states or trends [22]. The analysis also helps determine sex-specific aspects of sports performance (e.g., physical preparedness) and gives considerable information for coaching and training [14]. 

Unlike other disciplines in which the topic of differences between men and women has been already studied, research in CrossFit^®^ is still lacking. While the performance data significantly differed in strength- and endurance-oriented disciplines, scaling in CrossFit^®^ is unique and considers strength differences only when exercising with external loads. Our article aimed to analyze and compare athletes’ performances in the 2021 CrossFit Games^®^. Our second aim was to discover to what extent the applied scaling is related to general physical differences between male and female athletes.

## 2. Materials and Methods

We selected 40 women (age 27.8 ± 5.1, height 164.6 ± 4.5 cm, weight 66.3 ± 4.7 kg) and 40 men (age 27.2 ± 3.7, height 177 ± 5.5 cm, weight 88.9 ± 4.9 kg) who competed in the 2021 CrossFit Games^®^ for our analysis. Although we recruited 40 women and 40 men for our study, the number of participants varied for each event (mostly due to their injuries), as explained in the results section. During the competition, women and men athletes were cut based on rankings after events 9 and 10 to 30 and 20 athletes, respectively.

Qualifying for the CrossFit Games^®^ is specific in many aspects and cannot be compared to any other discipline. The criteria has changed several times in the past; here, we used the requirements for 2021 [25]. The first phase is the online initial round called CrossFit Open, in which anyone aged 14 and older can participate. It lasts for three weeks, each Thursday one event is announced, and the athletes must submit their scores within the following four days. The top 10% from each continent advance to the quarterfinals, which are also held online. In two days, the athletes take part in four events. Then, the best 30 athletes qualify for 10 in-person semifinals. From here, the best three advance to the CrossFit Games^®^. Then, there is a last-chance qualifier for all the athletes who missed the cut in the semifinals. They take part in four events and the best 10 also advance to the CrossFit Games^®^. A total of 40 men and 40 women take part in the Rx division of the CrossFit Games^®^. 

We extracted all data from the CrossFit Games^®^ official website and obtained information about the athletes from their profiles [25]. Other data—ranking, distribution of points, results (time, number of repetitions, lifted weight, etc.)—are shown on the leaderboard. We analyzed all results for each event. We evaluated the representation of modalities G, W, and M, and the scaling for each event, separately.

### 2.1. Performance

The CrossFit Games^®^ lasted for four days during which the athletes took part in 15 events (Table 1). After the 9th event, there was a cut to 30 athletes and after the 10th event, another cut to 20 athletes. The specific composition of the events is announced during the contest. Rankings (relative scoring system) and appropriate points are awarded according to event results (100 points for 1st place). Points distribution depends on the number of athletes—the last athlete obtains 1, 2, or 5 points after the last cut. The athlete with the highest total sum of points from all the events wins. 

There are two basic variations of events: you can either finish a given task in the shortest time possible *(the time variation)* or do as many repetitions as possible in the given time. There is a time cap for *the time variation.* Our research results consist of a specific time and the number of missing or completed repetitions (weight lifted). The athletes did not complete all repetitions within the time cap in six events (2, 4, 6, 7, 8, 14). We converted scores into repetitions per second to accurately process the results. Mangine et al. used the same procedure [10]. 

The events were designed according to the traditional CrossFit^®^ standards—the conditions for men and women in G and M were set as equal with the following exceptions: for event no. 5, the distance on Ski Erg was lowered to 400 m (−20%) in the women’s category. For event no. 6, the time caps were 7 and 6 min for men and women, respectively. The women’s load in W was scaled to 64–82% of men’s weight. The scale of more commonly used exercises (Olympic weightlifting, dumbbell) was between 64–72% in the women’s category.

The number of modalities (G, W, M) that a CrossFit performance comprises should be equal. In the case of the 2021 CrossFit Games^®^, it was 8x G, 11x W and 11x M. Although G is included fewer times, there were two events where G was a single modality, which makes the total number more balanced.

### 2.2. Statistical Analysis

Because this is an exploratory study, descriptive statistics and effect size were used for statistical processing. In descriptive statistics, the data are presented as the mean ± standard deviation (SD). To support the effect size results, differences between men and women were calculated as the percentage difference. The IBM SPSS 18.0.1 statistics program was used for data processing. As each event has its particular features, the data are presented as time averages (h: mm: ss), load averages (kg), and an average of the recalculation of the duration of each repetition (ss.ss). Cohen’s d with the scale <0.20 = trivial, 0.20–0.49 = small, 0.50–0.79 = medium, ≥0–80 = large was used to assess the material significance of performance differences [26]. This procedure was selected due to the independence of the sample size (i.e., the decreasing number of competitors during events), the characteristics of the effect size of the differences, as well as the context (i.e., the difference between men and women) [27].

## 3. Results

Athletes took part in 15 events that were different both in terms of their nature and duration. The average event durations were 14 min and 24 s for women and 13 min and 23 s for men (event no. 1 differed considerably in this regard, and when using the median, the overall result was 8 min and 6 s for women and 7 min and 26 s for men). The differences are 7.1% and 8.2% for women and men, respectively.

Table 2 shows the results for men and women in individual events, including absolute and percent differences. In six events, the athletes who failed to complete all repetitions in the time cap are in italics. Event no. 4 is the only one in which women achieved better results. The results are presented as time averages (hours: minutes: seconds) (events nos. 1, 3, 5, 9, 10, 13, 15), load averages (kg) (event no. 12), and the averages of the recalculation of the duration of each repetition (seconds) (events nos. 2, 4, 6, 7, 8, 12, 14).

For events no. 7 and 8, the difference between the results for men and women was 17.2% and 12.9%, respectively, although the effect size was d = −0.112 and d = 0.082, respectively. The reason is a large standard deviation; however, according to the comparison of averages, the difference can be considered meaningful.

There were only marginal differences between men and women in events no. 1, 5, 6, and 14 if we take the effect size and percentage into account. In event no. 5, contrary to other endurance disciplines, the distance on SkiErg in the women’s category was shortened.

The differences between event results ranged from 0.1% (event no. 6) to 33.1% (event no. 12). We observed the biggest difference between the sexes for the event that comprised weightlifting (1 RM snatch)—weights in the ladder were scaled. We observed the greatest statistical differences (large size effect) in events no. 2, 3, 9, 10, 11, and 12.

## 4. Discussion

Our study aimed to describe the performance differences between male and female athletes at the 2021 CrossFit Games^®^. We analyzed 15 events from the 2021 event and evaluated percentage differences. Women performed better in one of the events; furthermore, differences between men and women were not meaningful in five other events. The purpose of our study was to also compare final scores between men and women after scaling. Even though the data demonstrated that scaling reduces differences between men and women for absolute performance, men still performed better in most events.

Although CrossFit^®^ uses scaling, men and women did not obtain identical results. Women were more successful in event no. 4, which consisted of two exercises, thrusters, and wall walks, and it was most demanding on upper-body strength, with a difference of 13.1%. The barbell’s weight was scaled to 135 lbs (72% of men’s load). Women appeared to have better predispositions for such types of physical activity—a high dependence on arm muscle recovery, in particular on beta-oxidation, aerobic glycolysis, and lactate metabolism [13]. Men achieved better results in 14 out of 15 events, ranging from 0.1 to 33.1%. Nevertheless, the differences were not meaningful in three of these events, confirming that there are differences among men and women in their performances, even when scaling is applied [14,16,24].

The difference between sexes in the longest endurance event (swimming combined with kayaking) was 2.9%. In general, the difference between men and women decreases for longer (>60 min) tracks [17,24]. The main reason is probably the more effective beta-oxidation of fat among women [13]. A higher difference between the sexes was obvious in the 550-yard sprint (event no. 3)—12.1%. This may be due to muscle morphology, lower absolute muscle force, or lower power output [15].

We observed a small difference (d = 0.082, 12.9%) in performance between men and women in gymnastics event no. 8. The difference in bodyweight upper-body performance is generally higher (40–62%) [22,23]; however, one-time maximum repetition is usually tested in this event. Event no. 8’s duration (handstand walking course) was approximately 3–4 min; therefore, we can expect a lower difference. The differences between sexes are noteworthy even in exercises with a higher repetition count that demand endurance and depend on technical proficiency (not the strict form).

In event no. 12, which comprised only weightlifting (1 RM snatch), the difference between men and women was 33.1%. Scaling in CrossFit^®^ with load adjustments to 64–72% is well set according to weightlifting or powerlifting performance [19,20] and corresponds to the real strength differences in CrossFit athletes. Thanks to that, women and men can participate in the same events; however, the existence of performance sex differences comparable to powerlifting or Olympic weightlifting has been confirmed [19,28].

The scaling for W is adequate for carrying out the same strength task for both sexes. In general, no scaling is used for M and G, so performance differences (like in relative strength or track and field performance) [16,21] are expected. If the aim was to obtain similar results, these modalities would also have to be scaled. Shortening the distance by 20% in event no. 5 led to only a small difference between men’s and women’s results (d = 0.368). Scaling endurance disciplines (M) could be a good way to reduce result differences; however, finding the right scaling levels is difficult because the performances of men and women differ by 6–12% depending on the track and field discipline, and by an even smaller percentage in longer durations (>30 min) [14]. On the other hand, the current goal of scaling is not to achieve the same result for men and women but to maintain tasks and a similar (external and internal) load.

Most events include multiple modalities that do not have the same difficulty. That is why, for example in the combination of G, W, and M (events no. 5, 11, 13, 15), there is a difference of 4.8–18.7% (from small to large size effect). Due to this, for specific combinations of modalities, it is difficult to determine any general conclusions of performance differences.

The reasons for performance differences are probably not based solely on anatomical and physiological differences. More frequent participation in sports leads to better adaptation and can increase the number of athletes, from whom the best are selected [29]. Other variables could affect performance differences: training regimen (length, methods, volume, etc.), psychological mechanisms (pain resistance, motivation to win), body composition, or incidence of injuries.

Although scaling in CrossFit^®^ follows clear rules [3], there have been random exceptions in recent years. Unfortunately, the reasons for this remain unknown, and the organizers of the CrossFit Games^®^ are not bound by any prescribed rules for compiling events. This condition makes it difficult to analyze and interpret the results. The fact that each CrossFit^®^ competition is unique and the events do not repeat can be regarded as a study limitation, and it is possible that results from previous years could be different. Additionally, we must take into consideration that some athletes dropped out during the competition.

## 5. Conclusions

CrossFit^®^ is a sports discipline that uses original scaling for men and women. Our analysis results of CrossFit Games^®^ athletes show a sex performance difference from 0.1 to 33.1%. The biggest difference was in Olympic weightlifting (1 RM snatch). Similar to other sports disciplines, there were differences in event results between the sexes. Despite scaling, men generally achieved better results because of their anatomical and physiological differences. On the other hand, women achieved better results in one event, and in the other four, the differences were small. However, CrossFit Games^®^ events are always unique and the events rarely repeat; therefore, the results from another year could differ. As this study is the first to compare results between sexes for the CrossFit Games^®^, additional future studies should be conducted to confirm our results.

## Figures and Tables

**Table 1:** CrossFit Games^®^ 2021 events.

Event 1	Event 2	Event 3	Event 4
For time: 1-mile swim with fins 3-mile kayak	For time: 126-ft. sled drag, 180 | 220 lb. 5 Pig flips, 350 | 510 lb. 12 muscle-ups 12 bar muscle-ups 12 bar muscle-ups 12 muscle-ups 5 Pig flips 126-ft. sled drag Time cap: 12 min.	For time: 550-yard sprint Time cap: 4 min.	For time: 10-9-8-7-6-5-4-3-2-1 reps of: Wall walks Thrusters, 135 | 185 lb. (short bars) Time cap: 20 min
**Event 5**	**Events 6/7**	**Event 8**	**Event 9**
4 rounds for time: 4 rope climbs 500/400 ski erg Sandbag carry	5 rounds for time of: 250-m run 1 clean Women: 165 | 175 | 185 | 195 | 205 lb. Men: 245 | 265 | 285 | 305 | 315 lb. Time cap: 6/7 min. 5 rounds for time of: 200-m run out of the Coliseum 1 clean Women: 210 | 215 | 220 | 225 | 230 lb. Men: 325 | 335 | 340 | 345 | 350 lb. Time cap: 8 min	For time: Navigate the handstand walking course Time cap: 5 min.	21-15-9: Echo bike (cal.) Snatch, 75 | 105 lb. (short bars) Time cap: 8 min.
**CUT TO 20 ATHLETES** **Event 11**	**Event 12**	**Event 13**	**Event 14**
11-min. AMRAP: 1 pegboard ascent 7 single-arm dumbbell overhead squats, 50 | 70 lb. 15 heavy double-unders	1-rep-max snatch	4 rounds of: 20 GHD sit-ups 8 cheese curd burpees over the hay bale, 70 | 100 lb. 168-ft. yoke carry, 425 | 605 lb. 1-min. reset Time caps by round: 2 | 2 | 2 | 3 min	6-10-14 reps of: Deadlifts, 275 | 405 lb. (short bars) Freestanding handstand push-ups Time cap: 7 min.
**Event 15**	-	-	-
For time: 600-m row 90 chest-to-bar pull-ups 36-ft. back-rack walking lunge 36-ft. front-rack walking lunge 36-ft. overhead walking lunge 135 | 185 lb., short bar Time cap: 11 min.	-	-	-

Weights and time caps for women are listed first.

**Table 2:** Comparison of men’s and women’s results.

Event	Modality (G. W. M)	N Women	N Men	Women Average	SD (s)	Men Average	SD (s)	Difference	Difference %	Cohen’s d	Size of Effect
1	M	36	38	1:16:55	354.62	1:14:41	249.24	0:02:13	2.9	0.437	small
2	M.W	36	38	*10.36*	*1.2*	*8.62*	*1.07*	*1.74*	20.1	1.523	large
3	M	36	37	0:01:31	5.1	0:01:20	4.53	0:00:11	12.1	2.366	large
4	G. W	36	37	9.77 *	*1.29*	*11.23*	*5.44*	*1.47*	13.1	−0.372 *	small
5	G. W. M	35	36	0:13:17	57.57	0:12:58	44.22	0:00:19	2.4	0.368	small
6	W. M	34	35	*40.7*	*3.99*	*40.66*	*3.62*	*0.04*	*0.1*	0.01	small
7	W. M	34	34	*70.4*	*105.1*	*60.1*	*76.8*	*10.32*	*17.2*	−0.112 *	small
8	G	34	34	*8.74*	*13*	*7.74*	*11.2*	*1*	*12.9*	0.082	small
9	W. M	34	34	0:05:12	31.91	0:04:05	22.72	0:01:07	21.5	2.422	large
10	G. M	30	30	0:24:05	88.51	0:22:41	78.17	0:01:24	5.8	1.008	large
11	G. W. M	20	20	184.55 ‘	29.95	227.3 ‘	33.39	42.75	18.7	1.348	large
12	W	20	20	82.6 ^	4.32	123.3 ^	10.2	40.7	33.1	5.218	large
13	G. W. M	19	20	0:08:16	192.95	0:07:20	150.17	0:00:56	11.3	0.323	small
14	G. W	19	20	*10.86*	*3.4*	*10.65*	*3.4*	*0.2*	1.9	0.036	small
15	G. W. M	19	20	0:07:55	42.29	0:07:32	42.29	0:00:23	4.8	0.626	medium

G—Gymnastics; W—Weightlifting; M—Monostructural metabolic conditioning; *—better performance among women; ^^^—kg; ‘—repetitions; italics—event, where all athletes failed complete all repetitions.

## Data Availability

The data used in this research are available at: https://games.crossfit.com/leaderboard/games/2021?division=1&sort=0. (accessed on 1 September 2022).
